# Phosphodiesterase type-5 inhibitors for erectile dysfunction following nerve-sparing radical prostatectomy: A network meta-analysis

**DOI:** 10.1097/MD.0000000000023778

**Published:** 2021-02-26

**Authors:** Jie Yang, Zhong-Yu Jian, Jia Wang

**Affiliations:** a Department of Urology, Chengdu First People's Hospital; b Department of Urology, Institute of Urology (Laboratory of Reconstructive Urology), West China Hospital, Sichuan University, Guoxue Xiang, Chengdu, Sichuan, P.R.C; c West China Biomedical Big Data Center, Sichuan University, Chengdu, China.

**Keywords:** erectile dysfunction, medical treatment, phosphodiesterase type-5 Inhibitors, radical prostatectomy, sexual health

## Abstract

**Background:**

To verify which phosphodiesterase type 5 inhibitors (PDE5is) strategy is better for erectile dysfunction (ED) following nerve-sparing radical prostatectomy (NSRP).

**Methods:**

This systematic literature search was conducted in MEDLINE, Web of Science and Cochrane Central Register of Controlled Trials database to identify eligible studies from the startup of these databases to 1 November, 2019. The ED recovery rate was the main outcome. Traditional pair-wise meta-analysis and multivariate random-effects network meta-analysis (NMA) were performed to explore direct and indirect comparisons, respectively. The surface under the cumulative ranking (SUCRA) probabilities was used to evaluate the efficacy of treatments.

**Results:**

A total of 14 randomized controlled trials with four kinds of PDE5is were included. Further pooled evidence suggested that PDE5is followed by NSRP had a benefit for penile rehabilitation compared to placebo using traditional pair-wise meta-analyses. Our NMA showed that Avanafil 200 mg on demand might be most likely to be the best treatment option according to the first rank of SUCRA both in NMA (SUCRA 83.5) and sensitivity analysis (SUCRA 90.2).

**Conclusion:**

Avanafil 200 mg on demand has the highest probability of being the best intervention among PDE5is in treating ED following NSRP. However, more randomized controlled trials are needed to validate this in consideration of the published data regarding Avanafil is relatively small scale.

## Abstract 2

Supplemental Digital Content is available in the text

## 1 Introduction

Prostate cancer ranks first among the most commonly diagnosed cancers in the elderly population and may also be the most commonly non-cutaneous cancer in men. In 2014, in the USA it was estimated that 29,480 men died of prostate cancer.^[1]^ Radical prostatectomy is still widely accepted for treating early localized prostate cancer. However, erectile dysfunction (ED) is common after nerve-sparing radical prostatectomy (NSRP) notwithstanding the increasing experience in surgical techniques.^[2]^ Although several methods are used in penile rehabilitation after NSRP, the most common treatment is oral phosphodiesterase type 5 inhibitors (PDE5is) daily or on demand despite that the fact PDE5is is less effective in populations who suffer ED after NSRP compared with the general population.^[3]^

Tadalafil, Vardenafil, Sildenafil, and Avanafil are all used for penile rehabilitation following NSRP, and the efficacy of these PDE5is has been demonstrated by more than one pair-wise meta-analysis when compared with placebo.^[4–6]^ However, which strategy is better in clinical practice is still lacking evidence, thus making it difficult to provide a recommendation.

Due to the rarely head-to-head trials among different PDE5is treatments, it is difficult to answer such a question comprehensively using only the pair-wise meta-analysis method. Surprisingly, the network meta-analysis (NMA) method^[7]^ can indirectly compare these treatments with the same comparator, and can also compare different treatments by combining direct and indirect comparisons simultaneously without losing randomization in individual trials.

Therefore, the aim of this study was to explore which PDE5is strategy is better in the treatment of ED following NSRP. A systematic review will be conducted to screen randomized controlled trials (RCTs) for further analysis. Then, a two-stage meta-analysis, namely traditional pair-wise meta-analysis and random-effects NMA, will be performed to answer this question considering all available data from included RCTs.

## 2 Methods

This study was conducted in accordance with the Preferred Reporting Items for Systematic Reviews and Meta-Analyses statement.^[8]^

### 2.1 Search strategy

According to the Preferred Reporting Items for Systematic Reviews and Meta-Analyses guidelines, we conducted a systematic literature search in MEDLINE, Web of Science and Cochrane Central Register of Controlled Trials database to identify eligible studies from the startup of these databases to 1 November, 2019. Restricting to the human participants and English language study was the limitation of the search strategy. Population, Intervention, Comparison, Outcome and Study design (PICOS) principle was used to search. The MeSH terms and text words used in Medline were (((((“phosphodiesterase 5 inhibitors”[Mesh]) OR phosphodiesterase 5 inhibitors))) AND (“Erectile Dysfunction”[Mesh] OR Erectile Dysfunction))) AND ((((randomized OR random∗) OR ((Randomized Controlled Trial [Publication Type]) OR “Randomized Controlled Trials as Topic”[Mesh])))) AND (Prostatectomy [Mesh] OR Prostatectomy). Two independent reviewers performed the literature search. If disagreements appeared, two authors discussed the first. If they could not reach consensus, it was resolved by consulting with a senior author.

### 2.2 Eligible criteria

Studies included in systematic review that matched the eligible criteria were screened based on PICOS evidence listed below:

(1) Participants: patients after NSRP,
(2) Intervention: PDE5is treatment;
(3) Comparison: compared with the placebo or PDE5is;
(4) Outcomes: the efficiency evaluations;
(5) Study design: only RCTs were included in meta-analysis.

The following types of literature were excluded from our study: meta-analysis, review, editorials, letters, comments, case reports, congress reports, and meeting abstracts.

### 2.3 Data extraction and Quality Assessment

Two authors independently extracted the information from each eligible study. Disagreements were resolved by discussion first. The third senior author will arbitrate if the disagreements still exist after discussion. The detailed data extraction information was shown in Table 1. The primary outcome was ED recovery rate according to individual results based on the International Index of Erectile Function (IIEF) questionnaires including IIEF, IIEF-EF and IIEF-5. The quality of included trials was evaluated according to the previously reported guidelines and the judgments for each quality item were classified as three grades: “high,” “unclear,” and “low.”^[8,9]^ Two independent authors assessed the quality of study and disagreements were resolved as the same as describe above.

**Table 1:** Summary of included studies using oral PDE5Is for penile rehabilitation after nerve-sparing radical prostatectomy.

Study (year)	Country	Interventions (participants,n)	Primary inclusion criteria	Primary outcomes	Therapy (mo)
Montorsi F 2004	Italy, USA, Canada	Tadalafil 20 mg OD (102) Placebo (201)	Patients with ED (erection affects satisfaction with sexual intercourse consistently) 12 to 48 months after BNSRRP, age≤65	IIEF-EF, SEP-2 and SEP-3	3
Aydogdu O 2011	Turkey	Tadalafil 20 mg 3times/week (32) No use of Tadalafil (33)	BNSRRP, age≤65, preoperative IIEF-EF scores > 25, SEP questions 2-3 ‘yes’	IIEF-EF, SEP-2 and SEP-3	6
Montorsi F 2014	Europe, Canada	Tadalafil 5 mg daily (139) Tadalafil 20 mg OD (143) Placebo (141)	BNSRP, age<68, preoperative IIEF-EF scores > 21	IIEF-EF, SEP, CPL	9
Canat L 2015	Turkey	Tadalafil 20 mg OD (40) Tadalafil 20 mg 3times/week (38) No use of Tadalafil (34)	BNSRRP, Patients with moderate or severe ED prior to the surgery were excluded	IIEF-6	12
Mulhall JP 2016	Europe, Canada	Tadalafil 20 mg OD (139) Tadalafil 5 mg daily (142) Placebo (141)	BNSRP, age<68, preoperative IIEF-EF scores > 21	IIEF-EF	9
Brock G 2003	England, USA, Canada	Vardenafil 10 mg OD (140) Vardenafil 20 mg OD (147) Placebo (140)	Patients with ED 6 to 60 months after UNSRRP/BNSRRP	IIEF-EF, SEP-2 and SEP-3	4
Montorsi F 2008	Europe, USA, Canada, South Africa	Vardenafil 10 mg daily (could be decreased to 5 mg if required) (207) Vardenafil starting at 10 mg OD with the option to titrate to 5 mg or 20 mg (204) Placebo (206)	BNSRP, age≤65, preoperative IIEF-EF scores > 25	IIEF-EF	9
Bannowsky A 2012	Germany	Vardenafil 10 mg daily (12) Vardenafil 5 mg daily (12) Placebo (12)	UNSRRP, who had been sexually active before surgery	IIEF-5	12
Bannowsky A 2008	Germany	Sildenafil 25 mg daily (23) Placebo (18)	UNSRRP/BNSRRP, age 54-75	IIEF-5	13
Padma-Nathan H 2008	USA, France, Belgium, Australia	Sildenafil 50 mg daily (40) Sildenafil 100 mg daily (41) Placebo (42)	BNSRRP, age≤70, normal preoperative erectile function (score of IIEF-3 and IIEF-4 was at least 8)	IIEF-5(Q3 and Q4)	9
Pace G 2010	Italy	Sildenafil 50 mg (or 100mg) daily (20) No use of Sildenafil (20)	BNSRRP, age 50–70, preoperative IIEF > 25	IIEF	2
Pavlovich CP 2013	USA	Sildenafil 50 mg daily (50) Sildenafil 50 mg OD (50)	Minimally invasive NSRP, preoperative IIEF-EF > 25	IIEF-EF	12
Kim DJ 2016	USA	Sildenafil 50 mg nightly + Sildenafil 100 mg OD (47) Sildenafil 100 mg OD (47)	BNSRRP, preoperative IIEF-EF > 21	IIEF-EF	12
Mulhall JP 2013	USA	Avanafil 100 mg OD (99) Avanafil 200 mg OD (99) Placebo (100)	Patients with severe ED 6 months after BNSRP, age≤70, Patients with ED prior to the surgery were excluded	IIEF-EF, SEP-2 and SEP-3	3

a = same trial, BNSRRP = bilateral nerve sparing retropubic radical prostatectomy, BNSRR = bilateral nerve sparing radical prostatectomy, CPL = Change in penile length, ED = erectile dysfunction, IIEF-EF = international index of erectile function- erectile function domain, ICI = intracavernosal alprostadil, IUA = intraurethral alprostadil, NA = not available, NSRP = nerve sparing radical prostatectomy, OD = on demand, PFMT = pelvic floor muscle training, Q = question, RA-RP = robot-assisted radical prostatectomy, SEP = sexual encounter profile, UK = united kingdom, UNSRRP = unilateral nerve sparing retropubic radical prostatectomy, USA = united states of America, VED = vacuum erection device.

### 2.4 Statistical Analyses

At the first stage, a traditional pair-wise meta-analysis was performed. Dichotomous variables in our study were expressed as odds ratio (OR) with 95% confidence interval (CI). The *Q* test and *I*^2^ statistics were used to evaluate heterogeneity. *I*^2^ > 50% or *P* < .1 indicated significant heterogeneity.^[10]^ Referring to the previous studies,^[11]^ random-effect model via the DerSimonian and Laird method was applied regardless of whether heterogeneity was high or low.

In the second stage, an NMA with random-effects approach was applied as previously described.^[12]^ A network graph was plotted to show the network of the included comparisons. Global inconsistency and local inconsistency were tested using the Higgins and Dias model respectively. If the p value was greater than or equal to 0.05, a consistency model was performed. Otherwise an inconsistency model would be applied^[13,14]^ because of the high risk of inconsistency. The biggest contribution of this model is that it introduces inconsistent parameters into the model, thereby theoretically avoiding the impact of confounding factors on the results. Based on the results of NMA, surface under the cumulative ranking curves (SUCRA) probabilities which is a commonly reported method in NMA, was used to evaluate the efficacy of different treatments.^[7]^

Sensitivity analysis was performed by omitting the study mainly contributed to the inconsistency in the NMA. Publication bias and small-study effects were demonstrated by comparison-adjusted funnel plots. All data from our meta-analysis were analyzed by Review Manager 5.3 and Stata 14.

## 3 Results

Supplementary Figure S1 presented a visual flowchart of the search strategy. Finally, after excluding some literature,^[15,16]^ a total of 14 RCTs with four kinds of PDE5is were included in our systematic review.^[17–30]^Table 1 summarized the detailed information for studies eventually included in the study. Supplementary Figure S2 showed the overall pooled risk of bias assessment in 14 studies. Owing to the generally relatively low quality of reporting methodology in studies included in meta-analysis, the potential risk of bias should not be ignored for the majority of the RCTs.

### 3.1 Direct Comparision between PDE5is and Placebo

**Figure 1:**
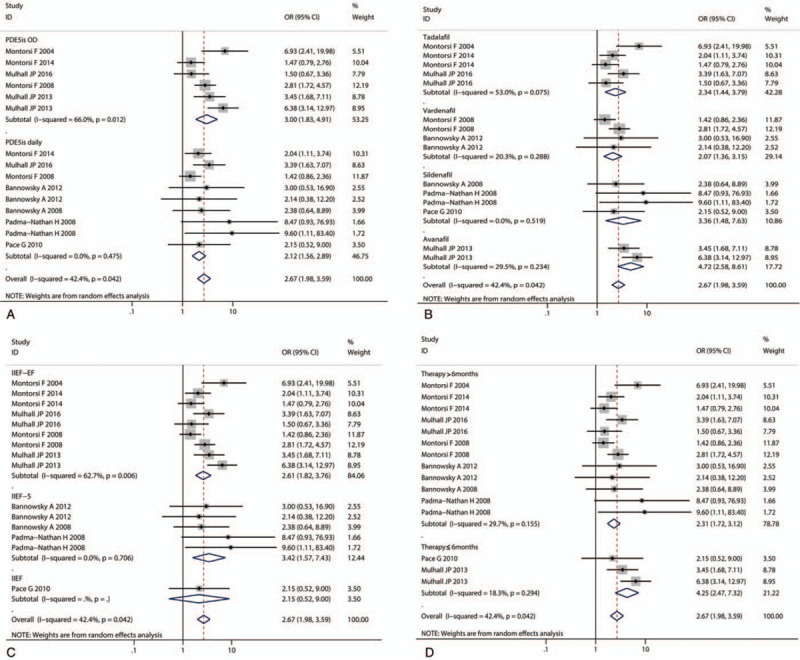
Pooled random effects OR (odds ratio) and 95% CI (confidence interval) for the erectile dysfunction recovery after nerve-sparing radical prostatectomy. Subgroup analyses based on the (A) PDE5is usage namely on demand (OD) or daily (nightly), (B) kinds of PDE5is (Tadalafil, Vardenafil, Sildenafil and Avanafil), (C) questionnaire categories and (D) therapy duration period.

Pooled evidence suggested that PDE5is followed by NSRP had a benefit for penile rehabilitation compared to placebo (OR = 2.67, 95%CI: 1.98, 3.59; *P* < .001) (Fig.1). We then performed subgroup analyses based on the PDE5is used, namely on demand (OD) or daily (nightly), kinds of PDE5is (Tadalafil, Vardenafil, Sildenafil and Avanafil), questionnaire categories, and therapy duration period. A stronger benefit was detected in the OD subgroup (OR = 3.00, 95%CI: 1.83, 4.91; *P* < .001) (Fig. 1A). The recovery rate in Tadalafil (OR = 2.34, 95%CI: 1.44, 3.79; *P* = .001), Vardenafil (OR = 2.07, 95%CI: 1.36, 3.15; *P* = .001), Sildenafil (OR = 3.36, 95%CI: 1.48, 7.63; *P* = .004), and Avanafil (OR = 4.72, 95%CI: 2.58, 8.61; *P* < .001) subgroups were all significantly higher compared to placebo (Fig. 1B). Similar results were obtained in both subgroup analyses according to the questionnaire categories (Fig. 1C) and the therapy duration time (Fig. 1D).

### 3.2 The results of NMA

**Figure 2:**
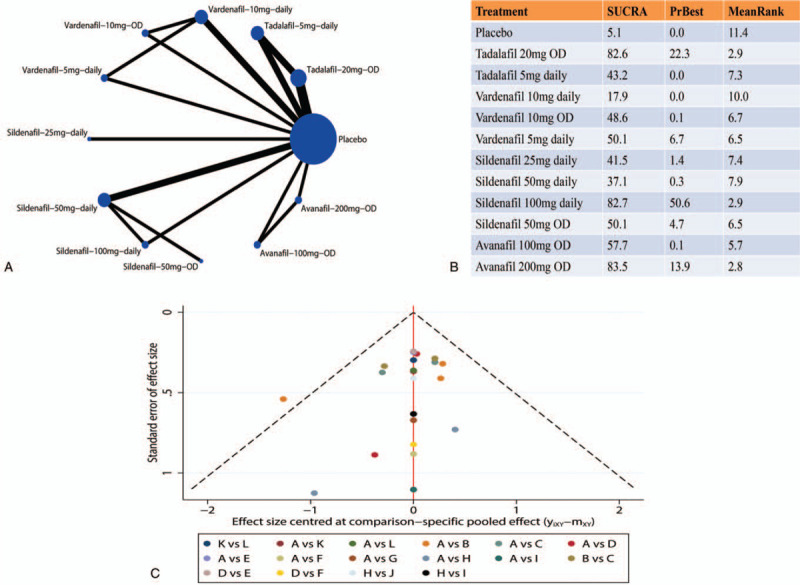
The results of network-meta analysis. (A) Network graph of comparison included in the analysis. Nodes are proportional to the number of patients, and edges are weighted according to the number of studies included in the comparisons. (B) The SUCRA of each regimen included in network meta-analysis. (C) Comparison-adjusted funnel plot of the studies included in this meta-analysis. (A = Placebo, B = Tadalafil-20mg-OD, C = Tadalafil-5mg-daily, D = Vardenafil-10mg-daily, E = Vardenafil-10mg-OD, F = Vardenafil-5mg-daily, G = Sildenafil-25mg-daily, H = Sildenafil-50mg-daily, I = Sildenafil-100mg-daily, J = Sildenafil-50mg-OD, K = Avanafil-100mg-OD, L = Avanafil-200mg-OD).

The network graph was shown in Figure 2A. The size of the nodes represented the proportion of participants. The edges were weighted based on the number direct comparison studies. The Higgins test showed that global inconsistency existed (chi^2^ = 7.95, Prob > chi^2^ = 0.047). In addition, local inconsistency, coming from loop comparison among Placebo-Tadalafil 20 mg OD-Tadalafil 5 mg daily, was also demonstrated (Supplementary Figure S3 and Supplementary Figure S4). Therefore, the following NMA was performed by inconsistency model,^[13,14]^ which introduced inconsistent parameters into the model, thereby theoretically avoiding the impact of confounding factors on the results.

Figure 2B showed the results of SUCRA enrolling all included studies. Avanafil 200 mg OD ranked first (SUCRA 83.5), thus having the highest probability of being the best intervention for improving ED recovery. The supporting material of the SUCRA, called the graph of the area under the curve was shown in Supplementary Figure S5.

### 3.3 Sensitivity analysis and publication bias

**Figure 3:**
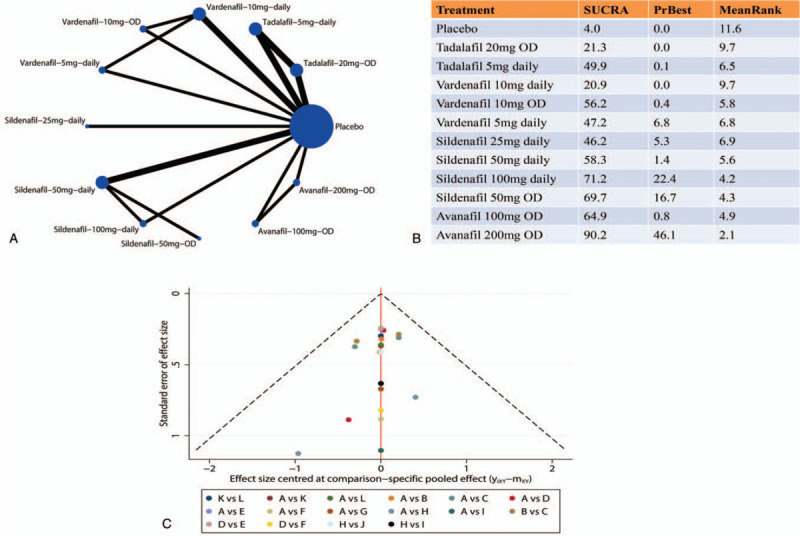
The results of sensitivity analysis. (A) Network graph of comparison included in the analysis. Nodes are proportional to the number of patients, and edges are weighted according to the number of studies included in the comparisons. (B) The SUCRA of each regimen included in network meta-analysis. (C) Comparison-adjusted funnel plot of the studies included in this meta-analysis. (A = Placebo, B = Tadalafil-20mg-OD, C = Tadalafil-5mg-daily, D = Vardenafil-10mg-daily, E = Vardenafil-10mg-OD, F = Vardenafil-5mg-daily, G = Sildenafil-25mg-daily, H = Sildenafil-50mg-daily, I = Sildenafil-100mg-daily, J = Sildenafil-50mg-OD, K = Avanafil-100mg-OD, L = Avanafil-200mg-OD).

The inconsistency in our NMA maily came from one study.^[17]^ After omitting this study in sensitivity analysis, both the global inconsistency (chi^2^ = 1.24, Prob > chi^2^ = 0.538) and local inconsistency (Supplementary Figure S6 and Supplementary Figure S7) were not statistically significant. Therefore, the consistency model was used for analysis and showed that Avanafil 200 mg OD (SUCRA 90.2) still had the highest probability of being the best treatment. And the graph of the area under the curve in sensitivity analysis was shown in Supplementary Figure S8. As shown in Figure 2C and Figure 3C, most of the plots were symmetrically distributed inside the 95% CIs in comparison-adjusted funnel plots which indicated a low risk of publication bias.

## 4 Discussion

Montorsi et al. first proposed penile rehabilitation in 1997.^[31]^ A large number of studies including clinical trials and meta-analyses have been reported on this topic. However, it is difficult for pair-wise meta-analysis to highlight this whole topic comprehensively because of the rarely head-to-head trials among different PDE5is treatments. To the best of our knowledge, this was the first NMA to verify which PDE5is strategy is better in the treatment of ED following NSRP.

There were three core assumptions that should be considered in the meta-analysis, including homogeneity, consistency and similarity. In our meta-analysis, differences in ED recovery definition, kinds of PDE5is, dosage, usage, and treatment time might mainly contribute to the homogeneity. To reduce the homogeneity, we tried our best effort to remove the differences in the types of PDE5is, dosage, and usage. However, the residual homogeneity could not be avoided. Owing to the random effect model performed, our results became more conservative by considering homogeneity. Publication bias was not significant in our study. However, inconsistency existed in NMA. An inconsistency model was applied,^[13,14]^ which introduced inconsistent parameters into the model, thereby theoretically avoiding the impact of confounding factors on the results. Surprisingly, there was no obvious inconsistency in the sensitivity analysis. In our study, similarity was also evaluated by detailed examination of each RCT. The quality of partly RCTs was relatively low under the guidance of the Cochrane library guideline. This led to the potential uncertainty risk of bias in our meta-analysis. In general, our results should be treated with caution.

Avanafil is a highly selective, quickly action and potent PDE5is that has been approved for the treatment for ED in general population.^[30]^ In our study, pooled evidence suggested that Avanafil 200 mg OD might be most likely to be the best treatment option for ED recovery after NSRP according to the first rank of SUCRA both in NMA (83.5) and sensitivity analysis (90.2). This result is in accordance with a previous meta-analysis, which reported that Avanafil on demand was the most effective PDE5is in EF recovery. In addition, our study found that Avanafil 200 mg OD might be more effective than Avanafil 100 mg OD. Due to the rapid improvements after administration,^[32]^ Avanafil OD seems to be more likely to be accepted by patients than planning to take it daily. However, it should be noted that the available evidence reporting Avanafil came from one trial.^[30]^ Although the quality of this trial was relatively high, a greater proportion of patients discontinued the study (24%) in the placebo group than the Avanafil group (11%). In addition, this trial was sponsored by industry. Therefore, the efficacy should be interpreted with caution.

Sildenafil is also a short-term PDE5i like Avanafil, the SUCRA of Sildenafil 100 mg daily was second only to Avanafil 200 mg OD in our NMA. In sensitivity analysis, Sildenafil 50 mg OD was comparable with Sildenafil 100 mg daily. Therefore, we recommend that Sildenafil 100 mg daily and Sildenafil 50 mg OD might be both suitable regimens for ED recovery after NSRP. However, these studies related to Sildenafil were small sample size. In addition, the methodological quality of the included studies reporting Sildenafil was relatively low. Other limitations, such as differences in patient selection and baseline score, existed for recommending Sildenafil as a treatment option. Therefore, this result should be treated with caution.

Our NMA suggested that SUCRA of two regimens, namly Vardenafil 10 mg OD (48.6) and Vardenafil 5 mg daily (50.1) took the middle place. In the following sensitivity analyses, the removal of data contributing to inconsistency did not change significantly. A previous meta-analysis reported that Vardenafil significantly improved ED recovery only when used OD.^[5]^ This bias was mainly caused by the different results of two included RCTs.^[23,24]^ In the multicenter, large sample and double-blind head-to-head comparison, Vardenafil OD treatment resulted in significantly greater recovery than Vardenafil daily used. Both showed better ED recovery compared with placebo.^[23]^ However, another single center and small sample RCT^[24]^ reported that there was no significant difference between Vardenafil daily (10 mg) and placebo. In the NMA, the placebo acted as the same comparator, so the OD and daily regimen seemed to have a comparable effect.

In total, more well-designed RCTs need to be enrolled in our analysis to answer this question. The same bias existed in the indirect comparison between Tadalafil. After the study mainly contributing to inconsistency in NMA was excluded,^[17]^ the sensitivity analysis in our study showed that Tadalafil 5 mg daily was more significant than Tadalafil 20 mg OD, which was consistent with the high quality RCT.^[19]^

Some limitations need to be addressed when interpreting our results. First, the evaluation questionnaires were not the same in our study. Second, the different treatment periods among single studies could cause heterogeneity and influence therapeutic efficacy. Third, patient inclusion criteria after NSRP differed would limit the systematic interpretation of the efficacy in the treatment of ED recovery. Fourth, due to certain methodological deficiencies and low quality RCTs, bias existed in our study. Finally, although we removed the differences in PDE5is compound, dosage and usage, the inconsistency and homogeneity caused by the factors mentioned above, including patient selection in the included individual study, baseline erectile score, and relatively low quality of partly RCTs could not be reduced.

## 5 Conclusion

This is the first NMA to explore which PDE5is strategy is better for ED recovery following NSRP. We found that all PDE5is were effective compared to placebo. Avanafil 200 mg OD has the highest probability of being the best intervention according to this network meta-analysis based on the currently available evidence. Thus, it could be recommended for these patients in clinical practice. However, several limitations should not be ignored as described above. Besides, in consideration of the published data regarding Avanafil in the ED after radical prostatectomy is relatively small scale, larger sample and well-designed head-to-head trials are needed to validate the role of Avanafil 200 mg OD for ED recovery after NSRP in the future.

## Author contributions

**Conceptualization:** Jie Yang, Zhong-Yu Jian, Jia Wang.

**Data curation:** Zhong-Yu Jian.

**Formal analysis:** Jie Yang, Zhong-Yu Jian.

**Funding acquisition:** Jia Wang.

**Investigation:** Jie Yang, Zhong-Yu Jian, Jia Wang.

**Methodology:** Jie Yang, Zhong-Yu Jian, Jia Wang.

**Project administration:** Jie Yang, Zhong-Yu Jian, Jia Wang.

**Resources:** Jie Yang, Zhong-Yu Jian, Jia Wang.

**Software:** Jie Yang, Zhong-Yu Jian, Jia Wang.

**Supervision:** Jie Yang, Zhong-Yu Jian, Jia Wang.

**Validation:** Jie Yang, Zhong-Yu Jian, Jia Wang.

**Visualization:** Jie Yang, Zhong-Yu Jian, Jia Wang.

**Writing – original draft:** Jie Yang.

**Writing – review & editing:** Zhong-Yu Jian, Jia Wang.

## Supplementary Material

Supplemental Digital Content

## Supplementary Material

Supplemental Digital Content

## Supplementary Material

Supplemental Digital Content

## Supplementary Material

Supplemental Digital Content

## Supplementary Material

Supplemental Digital Content

## Supplementary Material

Supplemental Digital Content

## Supplementary Material

Supplemental Digital Content

## Supplementary Material

Supplemental Digital Content

## References

[1] Siegel R Desantis C Jemal A. Colorectal cancer statistics, 2014. CA Cancer J Clin 2014;64:104–17. 24639052 10.3322/caac.21220

[2] Schauer I Keller E Muller A. Have rates of erectile dysfunction improved within the past 17 years after radical prostatectomy? A systematic analysis of the control arms of prospective randomized trials on penile rehabilitation. Andrology 2015;3:661–5. 26198796 10.1111/andr.12060

[3] Clavell-Hernandez J Ermec B Kadioglu A. Perplexity of penile rehabilitation following radical prostatectomy. Turk J Urol 2019;45:77–82. 30875285 10.5152/tud.2019.18488 PMC6368034

[4] Liu C Lopez DS Chen M. Penile rehabilitation therapy following radical prostatectomy: a meta-analysis. J Sex Med 2017;14:1496–503. 29122494 10.1016/j.jsxm.2017.09.020

[5] Limoncin E Gravina GL Corona G. Erectile function recovery in men treated with phosphodiesterase type 5 inhibitor administration after bilateral nerve-sparing radical prostatectomy: a systematic review of placebo-controlled randomized trials with trial sequential analysis. Andrology 2017;5:863–72. 28787547 10.1111/andr.12403

[6] Tian D Wang XY Zong HT. Efficacy and safety of short- and long-term, regular and on-demand regimens of phosphodiesterase type 5 inhibitors in treating erectile dysfunction after nerve-sparing radical prostatectomy: a systematic review and meta-analysis. Clin Interv Aging 2017;12:405–12. 28260869 10.2147/CIA.S122273 PMC5325109

[7] Mills EJ Thorlund K Ioannidis JP. Demystifying trial networks and network meta-analysis. BMJ 2013;346:f2914. 23674332 10.1136/bmj.f2914

[8] Liberati A Altman DG Tetzlaff J. The PRISMA statement for reporting systematic reviews and meta-analyses of studies that evaluate healthcare interventions: explanation and elaboration. BMJ 2009;339:b2700. 19622552 10.1136/bmj.b2700 PMC2714672

[9] Higgins JPT Green S. Cochrane handbook for systematic reviews of interventions version 5.1.4 (updated March 2011). 2012. Accessed 27 Feb, 2011.

[10] Higgins JP Thompson SG. Quantifying heterogeneity in a meta-analysis. Stat Med 2002;21:1539–58. 12111919 10.1002/sim.1186

[11] Hempel S Newberry SJ Maher AR. Probiotics for the prevention and treatment of antibiotic-associated diarrhea: a systematic review and meta-analysis. JAMA 2012;307:1959–69. 22570464 10.1001/jama.2012.3507

[12] Jian Z Chen Y Liu Q. Combination of solifenacin and tamsulosin may provide additional beneficial effects for ureteral stent-related symptoms-outcomes from a network meta-analysis. World J Urol 2019;37:289–97. 30030658 10.1007/s00345-018-2404-6

[13] Sturtz S Bender R. Unsolved issues of mixed treatment comparison meta-analysis: network size and inconsistency. Res Synth Methods 2012;3:300–11. 26053423 10.1002/jrsm.1057

[14] Song F Clark A Bachmann MO. Simulation evaluation of statistical properties of methods for indirect and mixed treatment comparisons. BMC Med Res Methodol 2012;12:138. 22970794 10.1186/1471-2288-12-138 PMC3524036

[15] Yavari K. Anti-Angiogenesis Therapy of Cancer Cells using 153Sm-Bevasesomab 2018;2:130–9.

[16] Hambardzumyan M Hayrapetyan A. Differential diagnosis of malignant melanoma and benign cutaneous lesions by ultrasound analysis. Sci Medicine Journal 2020;2:100–7.

[17] Montorsi F Nathan HP McCullough A. Tadalafil in the treatment of erectile dysfunction following bilateral nerve sparing radical retropubic prostatectomy: a randomized, double-blind, placebo controlled trial. J Urol 2004;172:1036–41. 15311032 10.1097/01.ju.0000136448.71773.2b

[18] Aydogdu O Gokce MI Burgu B. Tadalafil rehabilitation therapy preserves penile size after bilateral nerve sparing radical retropubic prostatectomy. Int Braz J Urol 2011;37:336–44. discussion 344-336. 21756381 10.1590/s1677-55382011000300007

[19] Montorsi F Brock G Stolzenburg JU. Effects of tadalafil treatment on erectile function recovery following bilateral nerve-sparing radical prostatectomy: a randomised placebo-controlled study (REACTT). Eur Urol 2014;65:587–96. 24169081 10.1016/j.eururo.2013.09.051

[20] Canat L Guner B Gurbuz C. Effects of three-times-per-week versus on-demand tadalafil treatment on erectile function and continence recovery following bilateral nerve sparing radical prostatectomy: results of a prospective, randomized, and single-center study. Kaohsiung J Med Sci 2015;31:90–5. 25645987 10.1016/j.kjms.2014.11.005 PMC11916535

[21] Mulhall JP Brock G Oelke M. Effects of Tadalafil Once-Daily or On-Demand vs Placebo on Return to Baseline Erectile Function After Bilateral Nerve-Sparing Radical Prostatectomy--Results from a Randomized Controlled Trial (REACTT). J Sex Med 2016;13:679–83. 27045264 10.1016/j.jsxm.2016.01.022

[22] Brock G Nehra A Lipshultz LI. Safety and efficacy of vardenafil for the treatment of men with erectile dysfunction after radical retropubic prostatectomy. J Urol 2003;170 (4 Pt 1):1278–83. 14501741 10.1097/01.ju.0000086947.00547.49

[23] Montorsi F Brock G Lee J. Effect of nightly versus on-demand vardenafil on recovery of erectile function in men following bilateral nerve-sparing radical prostatectomy. Eur Urol 2008;54:924–31. 18640769 10.1016/j.eururo.2008.06.083

[24] Bannowsky A van Ahlen H Loch T. Increasing the dose of vardenafil on a daily basis does not improve erectile function after unilateral nerve-sparing radical prostatectomy. J Sex Med 2012;9:1448–53. 22462626 10.1111/j.1743-6109.2012.02705.x

[25] Bannowsky A Schulze H van der Horst C. Recovery of erectile function after nerve-sparing radical prostatectomy: improvement with nightly low-dose sildenafil. BJU Int 2008;101:1279–83. 18284406 10.1111/j.1464-410X.2008.07515.x

[26] Padma-Nathan H McCullough AR Levine LA. Randomized, double-blind, placebo-controlled study of postoperative nightly sildenafil citrate for the prevention of erectile dysfunction after bilateral nerve-sparing radical prostatectomy. Int J Impot Res 2008;20:479–86. 18650827 10.1038/ijir.2008.33

[27] Pace G Del Rosso A Vicentini C. Penile rehabilitation therapy following radical prostatectomy. Disabil Rehabil 2010;32:1204–8. 20156044 10.3109/09638280903511594

[28] Pavlovich CP Levinson AW Su LM. Nightly vs on-demand sildenafil for penile rehabilitation after minimally invasive nerve-sparing radical prostatectomy: results of a randomized double-blind trial with placebo. BJU Int 2013;112:844–51. 23937708 10.1111/bju.12253

[29] Kim DJ Hawksworth DJ Hurwitz LM. A prospective, randomized, placebo-controlled trial of on-Demand vs. nightly sildenafil citrate as assessed by Rigiscan and the international index of erectile function. Andrology 2016;4:27–32. 26663669 10.1111/andr.12118

[30] Mulhall JP Burnett AL Wang R. A phase 3, placebo controlled study of the safety and efficacy of avanafil for the treatment of erectile dysfunction after nerve sparing radical prostatectomy. J Urol 2013;189:2229–36. 23219537 10.1016/j.juro.2012.11.177

[31] Montorsi F Guazzoni G Strambi LF. Recovery of spontaneous erectile function after nerve-sparing radical retropubic prostatectomy with and without early intracavernous injections of alprostadil: results of a prospective, randomized trial. J Urol 1997;158:1408–10. 9302132

[32] Goldstein I McCullough AR Jones LA. A randomized, double-blind, placebo-controlled evaluation of the safety and efficacy of avanafil in subjects with erectile dysfunction. J Sex Med 2012;9:1122–33. 22248153 10.1111/j.1743-6109.2011.02629.x

