# Supplementary figures and images for: Phosphodiesterase type-5 inhibitors for erectile dysfunction following nerve-sparing radical prostatectomy: A network meta-analysis
Source: Medicine (Baltimore). 2021 Feb 26;100(8):e23778. doi: 10.1097/MD.0000000000023778 (PMC7909136; doi:10.1097/MD.0000000000023778)

Figure S1：Flow chart showing the relevant studies included in analysis.


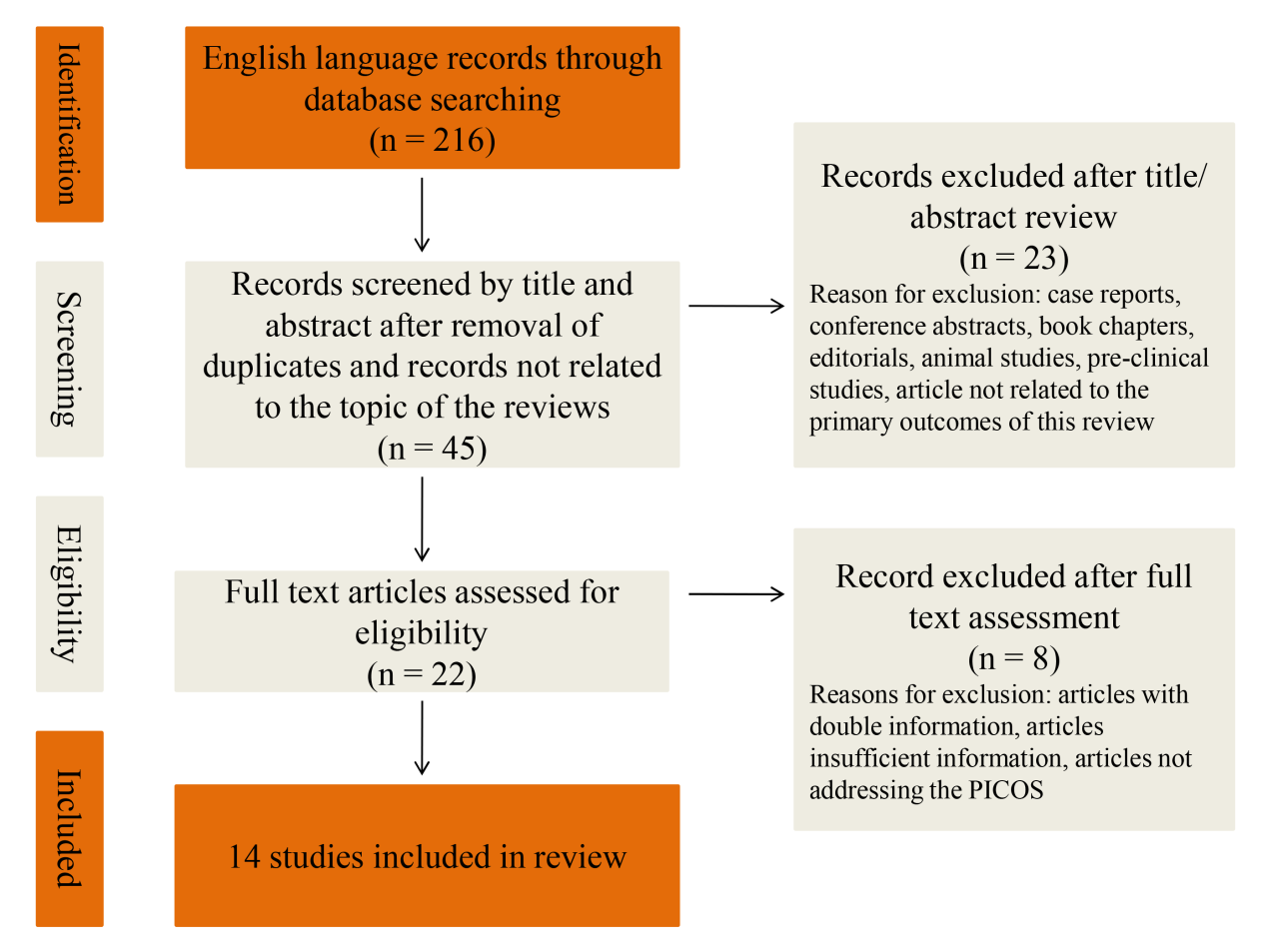

Supplement: Supplemental Digital Content [file medi-100-e23778-s001.docx]

Figure S2：Overall pooled risk of bias assessment of included studies.


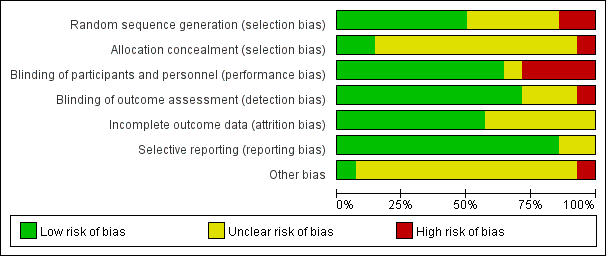

Supplement: Supplemental Digital Content [file medi-100-e23778-s002.docx]

Figure S4：The loop A-B-C (Placebo-Tadalafil 20mg OD-Tadalafil 5mg daily) showed inconsistency (IF = 1.55, 95%CI; 0.32, 2.78).


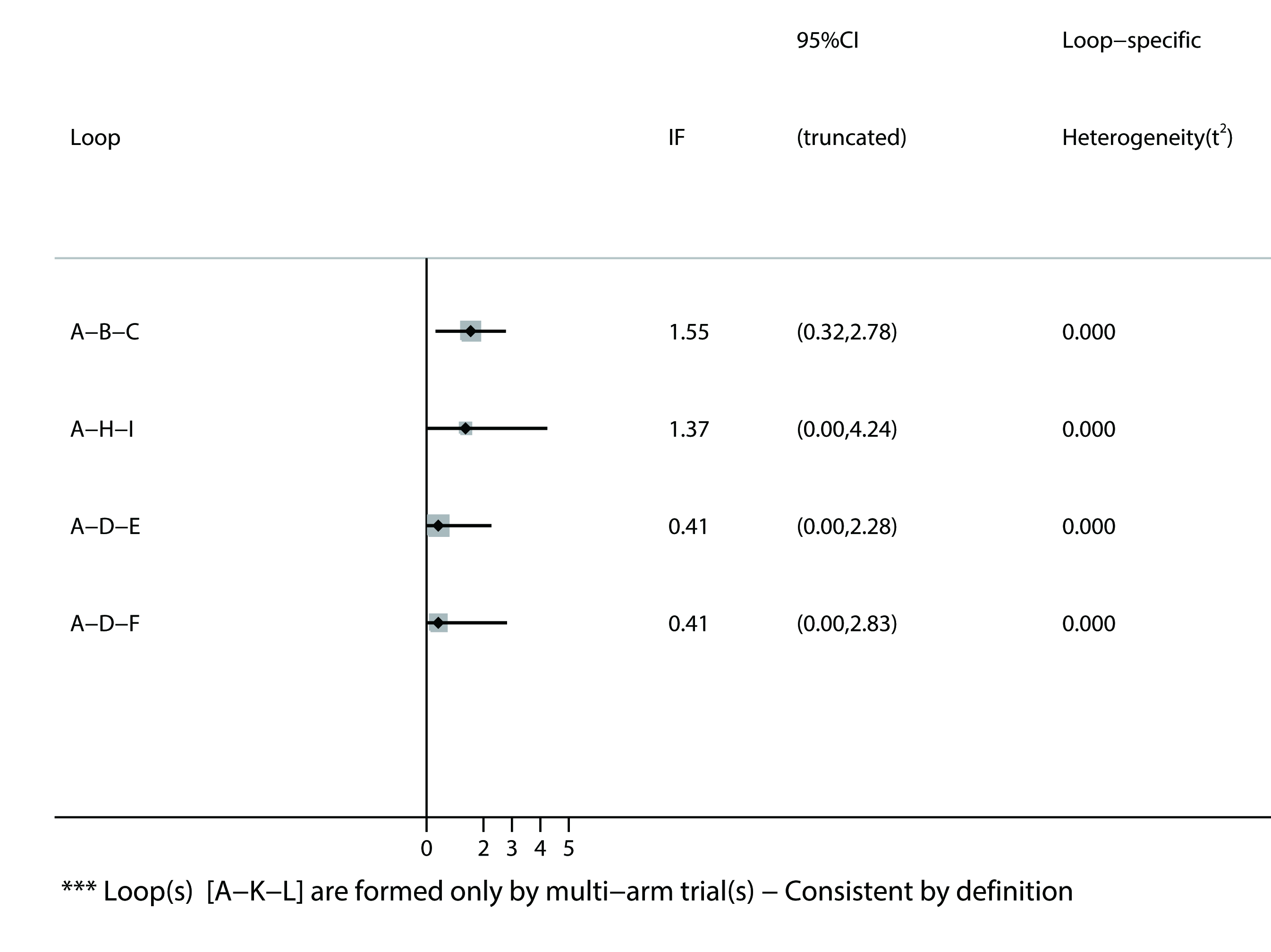

Supplement: Supplemental Digital Content [file medi-100-e23778-s004.docx]

Figure S7：No local inconsistency was obtained in sensitivity analysis.


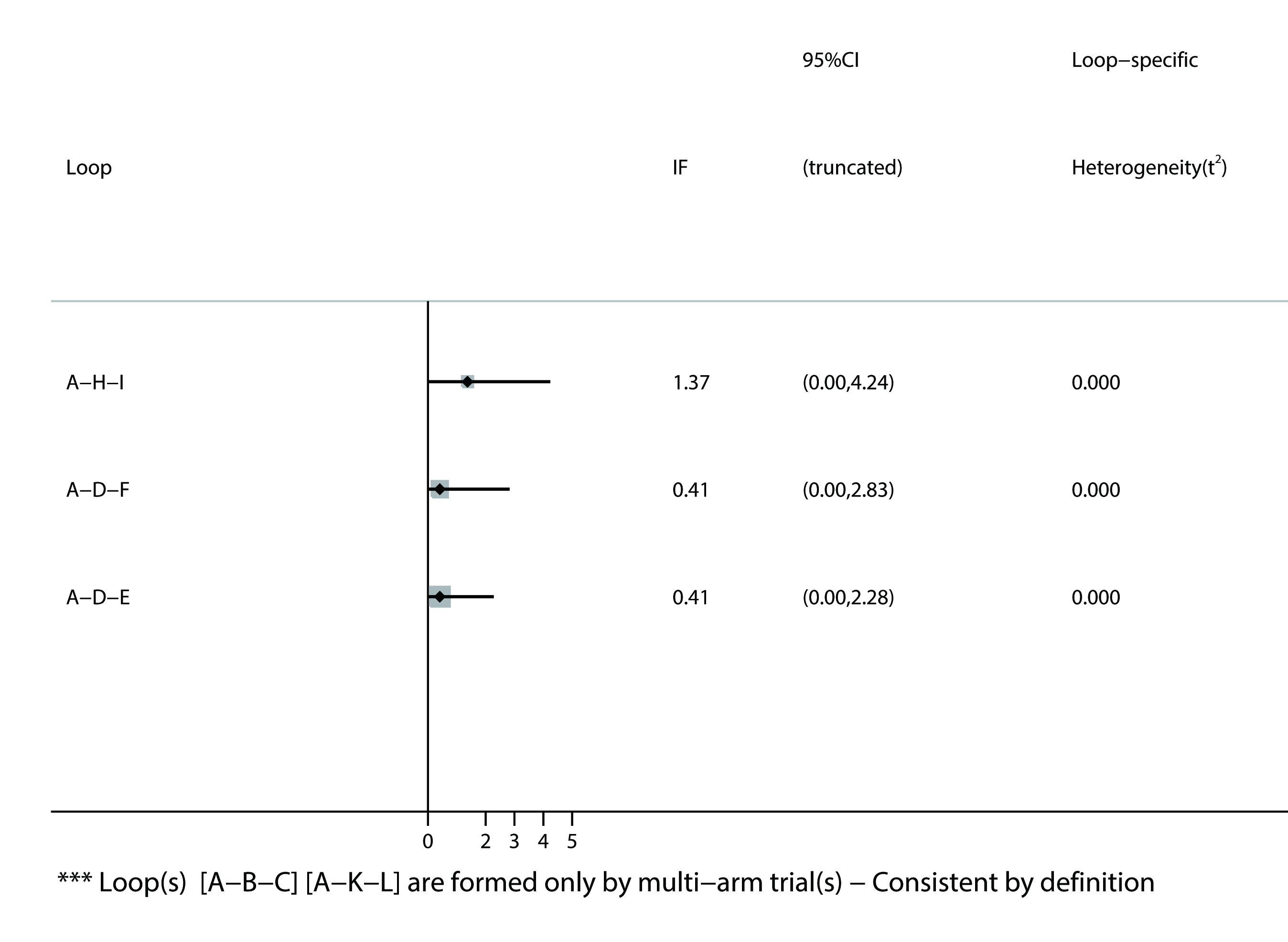

Supplement: Supplemental Digital Content [file medi-100-e23778-s007.docx]
