# Supplementary material for: Phosphodiesterase type-5 inhibitors for erectile dysfunction following nerve-sparing radical prostatectomy: A network meta-analysis
Source: Medicine (Baltimore). 2021 Feb 26;100(8):e23778. doi: 10.1097/MD.0000000000023778 (PMC7909136; doi:10.1097/MD.0000000000023778)

Figure S3：The node-splitting method showed inconsistency mainly in loop A-B-C (Placebo-Tadalafil 20mg OD-Tadalafil 5mg daily) (P = 0.010).


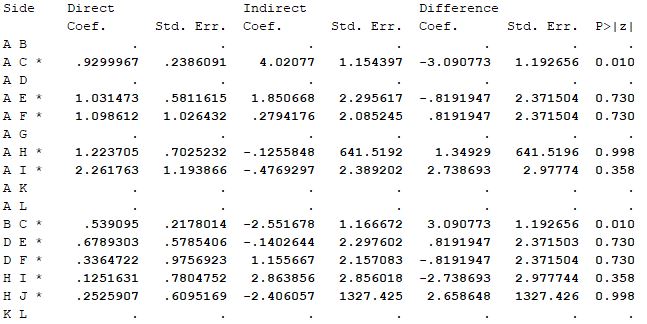

Supplement: Supplemental Digital Content [file medi-100-e23778-s003.docx]
