# Supplementary material for: Phosphodiesterase type-5 inhibitors for erectile dysfunction following nerve-sparing radical prostatectomy: A network meta-analysis
Source: Medicine (Baltimore). 2021 Feb 26;100(8):e23778. doi: 10.1097/MD.0000000000023778 (PMC7909136; doi:10.1097/MD.0000000000023778)
Supplement: Supplemental Digital Content [file medi-100-e23778-s008.docx]

Figure S8：The graph of SUCRA rank in sensitivity analysis. The area under the curve represents the ranking of SUCRA.
